# Ruptured Kommerell Diverticulum and Right-Sided Aortic Arch Treated Using a Non–Patient-Specific Custom-Made Triple Arch Branched Endograft

**DOI:** 10.1016/j.atssr.2025.02.010

**Published:** 2025-03-08

**Authors:** Michele Piazza, Piero Battocchio, Elda Chiara Colacchio

**Affiliations:** 1 Vascular and Endovascular Surgery Section, Department of Cardiac, Thoracic, Vascular Sciences and Public Health, University of Padova, Padova, Italy

## Abstract

We describe this unique case of a ruptured Kommerell diverticulum treated by adapting a stock-available triple-branched custom-made endograft, nonpatient specific, and bilateral carotid-to-subclavian bypass. The final angiography and the postoperative computed tomographic angiography showed the exclusion of the rupture with excellent endovascular geometric reconstruction and regular patency of all supra-aortic trunks. The patient was extubated on postoperative day 3, and the clinical course was uneventful. This report demonstrates that in extremely selected cases, the adaptation of a non–patient-specific custom-made branched device is feasible and may guarantee excellent results in emergency settings.

Endovascular repair of the aortic arch provides different solutions, and 3-vessels inner-branch endografts have shown good results in large series, with mortality and morbidity comparable to open repair.^1^ We present a case of a ruptured Kommerell diverticulum (KD) with a right-sided aortic arch.

A 47-year-old man presenting to the emergency department with chest pain and hypotension was diagnosed with a ruptured KD involving the origin of the left aberrant subclavian artery (LASA). The patient had a severely angulated type III^2^ right-sided aortic arch, with the left common carotid artery (CCA) originating first in the ascending aorta, followed by the right CCA, the right subclavian artery (RSA), and then the LASA (Figure 1). Both CCAs had an average diameter of 6.5 mm.

**Figure 1:**
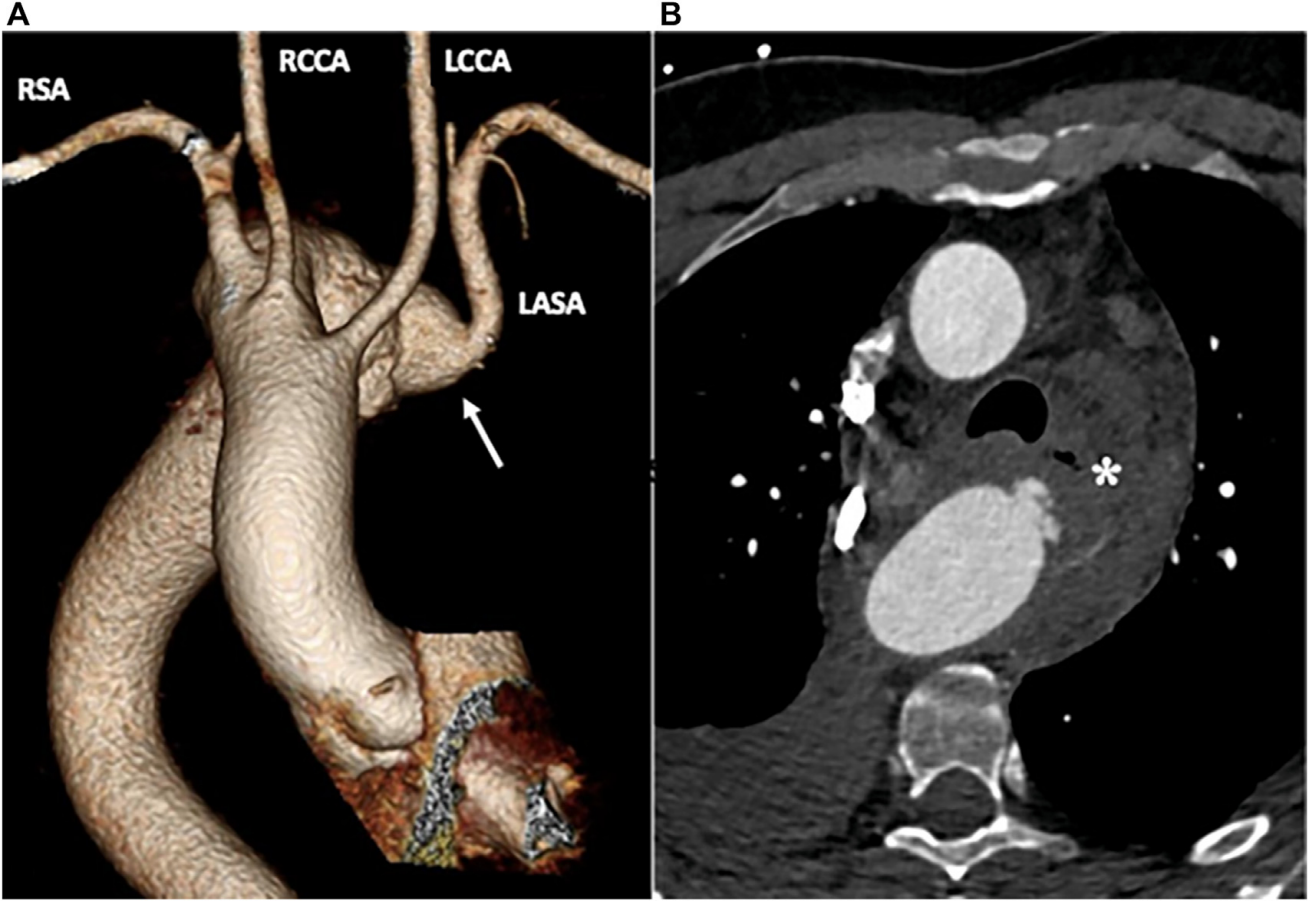
(A) The patient’s aortic arch anatomy, with (B) the Kommerell diverticulum (white arrow) and its rupture (white asterisk). (LASA, left aberrant subclavian artery; LCCA, left common carotid artery; RCCA, right common carotid artery; RSA, right subclavian artery.)

We adapted to this urgent case a stock-available custom-made 3-vessels branched stent graft (William Cook Europe, Bjaeverskov, Denmark) that was designated to another patient.

The procedure was performed under general anesthesia, in a hybrid operating room under fusion imaging guidance. First, we performed the left carotid-to-subclavian bypass using an 8-mm polytetrafluoroethylene graft (Gore Medical, Flagstaff, AZ). Through a right transfemoral percutaneous access, after a guidewire was placed across the aortic valve into the left ventricle (SAFARI^2^, Boston Scientific, Marlborough, MA), the self-oriented triple branched endograft (William Cook Europe) was advanced and fully deployed under rapid pacing and fusion imaging starting from the ascending aorta. The third-retrograde branch preloaded system was retrieved, and the graft shaft was removed.

Through a direct puncture of the left CCA, the first inner-branch was cannulated, and a 13 × 10 mm + 10 × 5 mm Gore Viabahn endoprosthesis (Gore Medical) was deployed to connect the branch cuff to the left CCA. The stent was reinforced with a 14 × 40 mm self-expanding nitinol bare stent (Epic, Boston Scientific) in the angled course and then dilated. At this point, we performed the right carotid-to-subclavian bypass also using an 8 mm polytetrafluoroethylene graft (Gore Medical). Then, the second inner branch was cannulated by a direct puncture of the right CCA and an 8 × 59 mm Gore Viabahn balloon-expandable endoprosthesis (Gore Medical) was advanced and deployed.

The third retrograde inner branch was cannulated from the femoral access, and a 10F Flexor introducer (Cook Medical, Bloomington, IN) was advanced through the branch into the KD. Through this access, both subclavian arteries were cannulated in an antegrade fashion, and embolized with two 14 mm Amplatzer Vascular Plugs (AVP; Abbott, Chicago, IL) for the RSA and a 16mm AVP for the LASA. Another 16 mm AVP was released in the aneurysm sac.

We then advanced a Gore TAG Conformable thoracic stent graft (Gore Medical) that was deployed proximally covering the third branch, and landing distally in the descending thoracic aorta to achieve an adequate distal sealing. Finally, we proceeded with the molding of the second thoracic component, crushing the third inner branch to the main graft wall.

The final digital subtraction angiography (Figure 2) and cone-bean computed tomography showed the complete exclusion of the ruptured KD with regular patency of the branched stent graft, and of both bypasses and carotid and subclavian arteries. The postoperative computed tomography angiography performed after 3 days from surgery confirmed these findings (Figure 3; Supplementary Video), with a successful clinical recovery of the patient, who was discharged after 7 days.

**Figure 2:**
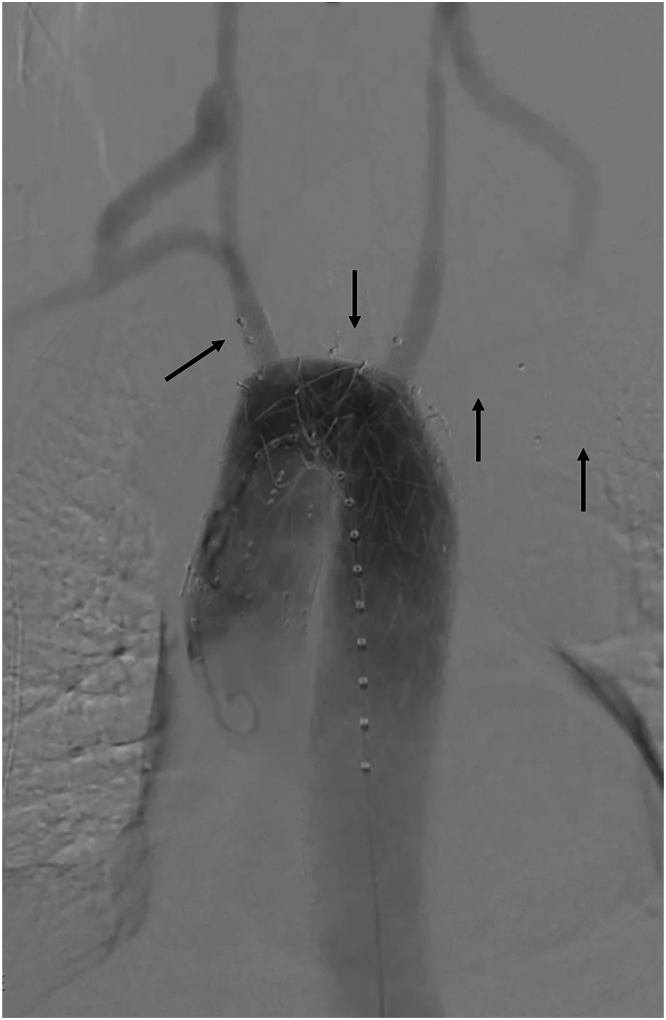
The final angiography shows the patency of the endograft and of the bypasses, with the exclusion of the rupture. The black arrows highlight the vascular plugs.

**Figure 3:**
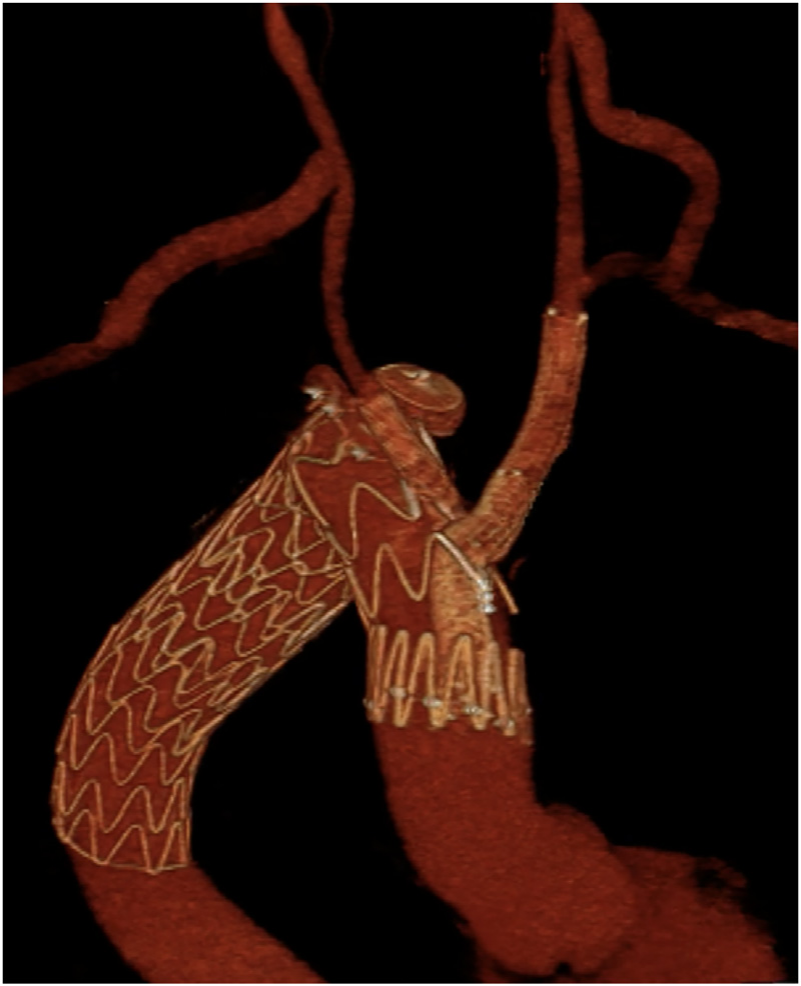
Three-dimensional reconstruction of the endograft implant shows the patency of supra-aortic trunks and bypasses and the exclusion of the aneurysm.

## Comment

Endovascular treatment of a ruptured KD associated with a right-sided aortic arch is reported in literature, often with the deployment in zone 2 of a tubular endograft only covering the origin of the LASA. However, the distal migration of the graft during the touch-up of the proximal landing zone^3^^,^^4^ is described, raising issues related to the stability of a thoracic endovascular aortic repair performed in zone 2 of the aortic arch. The only case we found in literature describing the treatment of a ruptured KD by deploying an endograft in the ascending aorta was presented by Yamashiro and colleagues,^5^ followed by debranching of the supra-aortic trunks, and the proximal anastomosis of a 4-branched surgical graft was realized in zone 0 through a median sternotomy and requiring aortic side clamping. Differently from the previous 2 reports,^3^^,^^4^ the thoracic stent graft did not migrate distally.

In our case, choosing to release this particular endograft in zone 0 provided increased stability to the whole implant and adequate effective proximal seal,^6^ with the 2 antegrade inner branches providing direct flow to both carotid axes. An alternative would have been landing with a thoracic endograft just after the left carotid artery, performing a left carotid-to-right subclavian bypass and right CCA reimplantation, without revascularization of the LASA: the entire brain perfusion would have relied on the single 6.5-mm left CCA, whose diameter is relatively small.

The custom arch endograft had compatible features to the patient’s anatomy because of the adequate graft oversizing and because the 2 antegrade inner branches were suitable for connection with the left carotid artery and right carotid artery. The left carotid-to-subclavian bypass was performed before the endograft deployment because it would have guaranteed a regular perfusion of at least the left carotid axis through the third retrograde branch in case of failure of the self-oriented mechanism of the endograft, with the branches deployed completely twisted in the inner curve of the arch rather that in the outer, thus covering the right carotid artery, left carotid artery, and RSA. Removing the third branch preloaded system after the full graft deployment would have allowed the rapid advance of a second distal thoracic tubular endograft proximally to the third branch, thus crushing it and closing the direct access to the rupture in case of hemodynamic instability.

The third retrograde branch was not used to revascularize one of the subclavian arteries because of the high risk of branch short-term instability. However, both subclavian arteries and the aneurysm sac were embolized through the third branch, with no need for arm access, thus shortening the intervention.

In our experience, adapting a non–patient-specific endograft to a complex arch anatomy in an emergent case of ruptured KD with a right-sided aortic arch is a viable solution with excellent short-term results. Detailed preoperative planning is mandatory, and limitations are related to the availability of custom arch branched devices, which is possible only in high-volume centers with large elective activity of endovascular arch repair.

## References

[1] Tenorio E.R., Oderich G.S., Kölbel T. (2021). Multicenter global early feasibility study to evaluate total endovascular arch repair using three-vessel inner branch stent-grafts for aneurysms and dissections. J Vasc Surg.

[2] Czerny M., Schmidli J., Adler S. (2019). Current options and recommendations for the treatment of thoracic aortic pathologies involving the aortic arch: an expert consensus document of the European Association for Cardio-Thoracic surgery (EACTS) and the European Society for Vascular Surgery (ESVS). Eur J Cardiothorac Surg.

[3] Motoki M., Hattori K., Kato Y. (2013). Endovascular repair of ruptured aberrant left subclavian artery with right aortic arch. Ann Thorac Surg.

[4] Takagi S., Goto Y., Yanagisawa J., Ogihara Y., Okawa Y. (2024). Thoracic endovascular aortic repair without subclavian revascularization of a ruptured Kommerell diverticulum. JACC Case Rep.

[5] Yamashiro S., Nagano T., Kuniyoshi Y., Kise Y., Maeda T., Arakaki R. (2012). Endovascular repair of intrathoracic ruptured Kommerell’s diverticulum. Asian Cardiovasc Thorac Ann.

[6] Piazza M., Squizzato F., Xodo A. (2021). Determination of optimal and safest proximal sealing length during thoracic endovascular aortic repair. Eur J Vasc Endovasc Surg.

